# Is There a Role for Absorbable Metals in Surgery? A Systematic Review and Meta-Analysis of Mg/Mg Alloy Based Implants

**DOI:** 10.3390/ma13183914

**Published:** 2020-09-04

**Authors:** Cortino Sukotjo, Tiburtino J. Lima-Neto, Joel Fereira Santiago Júnior, Leonardo P. Faverani, Michael Miloro

**Affiliations:** 1 Department of Restorative Dentistry, College of Dentistry, University of Illinois at Chicago, Chicago, IL 60612, USA; 2 Oral and Maxillofacial Surgery, Department of Diagnosis and Surgery, Division of Oral and Maxillofacial Surgery, School of Dentistry, São Paulo State University—Unesp, Araçatuba, São Paulo 16015-050, Brazil; tiburtinoneto@hotmail.com; 3 Department of Health Sciences, Centro Universitário Sagrado Coração-UNISAGRADO, Bauru, São Paulo 16011-160, Brazil; jf.santiagojunior@gmail.com; 4 Department of Diagnosis and Surgery, Division of Oral and Maxillofacial Surgery and Implantology, School of Dentistry, São Paulo State University—Unesp, Araçatuba, São Paulo 16015-050, Brazil; leonardo.faverani@unesp.br; 5 Department of Oral and Maxillofacial Surgery, College of Dentistry, University of Illinois at Chicago, Chicago, IL 60612, USA; mmiloro@uic.edu

**Keywords:** bone surgery, absorbable implants, magnesium (Mg), oral and maxillofacial, orthopedic, titanium (Ti)

## Abstract

Magnesium (Mg) alloys have received attention in the literature as potential biomaterials for use as absorbable implants in oral and maxillofacial and orthopedic surgery applications. This study aimed to evaluate the available clinical studies related to patients who underwent bone fixation (patients), and received conventional fixation (intervention), in comparison to absorbable metals (comparison), in terms of follow-up and complications (outcomes). A systematic review and meta-analysis were performed in accordance with the PRISMA statement and PROSPERO (CRD42020188654), PICO question, ROBINS-I, and ROB scales. The relative risk (RR) of complications and failures were calculated considering a confidence interval (CI) of 95%. Eight studies (three randomized clinical trial (RCT), one retrospective studies, two case-control studies, and two prospective studies) involving 468 patients, including 230 Mg screws and 213 Titanium (Ti) screws, were analyzed. The meta-analysis did not show any significant differences when comparing the use of Mg and Ti screws for complications (*p* = 0.868). The estimated complication rate was 13.3% (95% CI: 8.3% to 20.6%) for the comparison group who received an absorbable Mg screw. The use of absorbable metals is feasible for clinical applications in bone surgery with equivalent outcomes to standard metal fixation devices.

## 1. Introduction

One of the most significant public health concerns is the high incidence of traumatic accidents resulting in skeletal injuries with the need for bone reduction and fixation [1,2,3,4,5]. These traumatic events significantly affect the quality of life of these accident victims. Elderly patients and those suffering from chronic systemic conditions, such as diabetes, osteoporosis, and other bone metabolic disorders, have an increased potential for poor outcomes and worse complications from the management of these injuries. In orthopedic and oral and maxillofacial surgical specialties, plates and screws are used to stabilize the fractured bone fragments [4,6,7,8,9], and the most commonly used material is commercially pure Ti (CPTi) and its alloys, especially Ti grade 5 (Ti-6Al-4V). Other metals have also been used, for example, cobalt-chromium-molybdenum alloys, as well as stainless steel. These are considered biocompatible materials and possess the mechanical resistance necessary to prevent bony segment mobility and allow for primary bone healing and revascularization between the bone fracture segments. However, investigators may have misunderstood the concept of inertia of the metals implanted into the human body over the past few decades, since some metals used for bone fixation purposes are nobler than others. However, after implantation, these metals are subjected regularly to mechanical, electrochemical, and temperature alterations, which have resulted in complications, such as infection, metal hypersensitivity, and foreign body reactions [10,11,12,13,14,15,16].

Furthermore, metal plates and screws used for permanent implantation for bone fixation may have issues when placed in growing children with disturbances in normal growth patterns. Therefore, to resolve these problems, absorbable materials were developed, with commercially-available polymers and co-polymer materials(polyglycolic and polylactic acids) manufactured into bone plates and screws. After fracture repair and completion of the bone healing process (at approximately six months), the resorbable fixation devices begin to degrade into carbon dioxide and water; therefore, a second stage surgery is not needed to remove the plates and screws after healing is complete. However, many questions still remain regarding the use of resorbable fixation devices since they possess lower mechanical resistance than conventional metal devices. In addition, there is difficulty in bending (molding) the resorbable plates during surgery using heated water or ultrasonic methods, the absence of radiopaque implants on post-surgical radiologic evaluation, and unpredictable tissue responses with possible bone resorption due to the process of acidic degradation of the co-polymer materials, such as poly-l-lactic acid [17,18,19,20].

The concept of absorbable metals has been developed recently to reduce these possible complications [21,22,23]. Mg and zinc-based degradable metal alloys were recently developed since these metals possess desirable characteristics such as adequate strength (tensile, bending, and torsional) for bone fracture fixation. The final degradation product is not acidic as with poly-l-lactic acid materials(PLLA). In vitro and in vivo investigations have been performed and have led to improvements in the biocompatibility, bone healing properties, and corrosion resistance of the absorbable metals [24,25,26]. The Zn-based alloys have been investigated, primarily due to their excellent electrochemical process [21,27], which does not result in the accumulation of gas cavities such as hydrogen [28]. Both metals have shown excellent biocompatibility during the degradation process. They were both safely metabolized, including simulation for osteoblastogenesis in bone surgeries [28,29]. However, pure Zn does not have enough mechanical properties for osteosynthesis materials for use in bone fracture fixation. The primary element used to increase its strength is copper (Cu), which results in a suitable alloy for use (ZnCu) [18,28]. However, it is no longer an absorbable metal [30]. Further, Zn-based alloys have only been used for cardiovascular stents thus far [30,31]. Therefore, for this review, we focus on the clinical outcomes of the Mg and Mg-based alloy implants only.

Regarding the clinical applications, recently, the literature has shown exemplary behavior of Mg alloys used for bone fracture fixation. In Germany, the first report was published using screws from Mg alloys to fixate hallux fractures [11,32,33]. Any decision-making related to a clinical situation should be performed after obtaining an acceptable level of scientific evidence. Based on this principle, this study aims to answer the research question: “Is there evidence to support the clinical application of absorbable metals for bone fixation, particularly Mg/Mg alloy based implants?” This systematic review aims to evaluate clinical studies related to patients who underwent bone fixation (patients). Clinical data on patients who received conventional non-resorbable metal plates or screws (intervention, e.g., Ti) will be compared to data on absorbable plates and screws (comparison). Biological outcomes and clinical follow-up, as well as complications (outcomes) will also be investigated [10,34]. Therefore, to understand the clinical behavior of these materials, this systematic review and meta-analysis will gather information from clinical studies regarding these issues. Additionally, in vivo studies are included to demonstrate the biological responses of these materials.

## 2. Materials and Methods

### 2.1. Standard Criteria and Type of Study

This systematic review followed the Cochrane criteria [35,36], and the PRISMA-P and PRISMA Statement [37,38] on systematic review and meta-analysis.

#### 2.1.1. Protocol and Registration

The researchers registered this systematic review in the PROSPERO database, under submission: CRD42020188654 entitle “Is there viability in the use of absorbable metals in bone surgery? Systematic Review and Meta-analysis”. The authors followed the PRISMA-P protocol for planning a systematic review [37].

#### 2.1.2. Eligibility Criteria

The researchers performed the analyses based on the PICO index:

1. Population: Patients undergoing surgical treatment of bone fractures or deformities with fixation devices.
2. Intervention: Fixation using conventional metal plates and/or screws, such as Ti alloys.
3. Comparison: Fixation using absorbable metal plates and/or screws (Mg).
4. Outcome: Survival rates of the fixation systems, systemic complications, pain scale, quality of life, and functional analysis.

### 2.2. Inclusion/Exclusion Criteria

#### 2.2.1. Inclusion Criteria

The studies were selected according to the search strategy with the following inclusion criteria: (1) English language; (2) clinical follow-up studies of at least 6 months including the following study types: retrospective, prospective, and controlled and randomized clinical trial (RCTs); (3) publication period analysis until 20 June 2020; (4) adults and children with no upper or lower age limit; (5) consecutive cases including over 5 patients.

#### 2.2.2. Exclusion Criteria

The exclusion criteria included: studies related to in vitro methodology; animal studies; studies with less than 5 patients or with incomplete data; studies only related to the absorbable plates and/or screws from PLLA; review papers; studies that did not allow the collection of the required information.

### 2.3. Study Search Strategy

The databases used were: Medline/PubMed; Cochrane Library; EMBASE. These searches were carried out for articles published until 20 June 2020. Additional contact was made with the authors when it was not possible to locate the article via the national online system or COMUT.

### 2.4. Searches

The keywords based on MeSH/PubMed were: Surgery, Bone Plates, Absorbable implants. The articles were selected on the following bases (Cochrane, 46 articles; Embase, 517 articles; Pubmed, 556 articles; total = 1119 articles).

A manual search was also carried out in the specific journals in the area: Biomedical Engineering, Foot Ankle Surgurgery, Musculoskelet Disorders, Journal of Orthopaedic Science, Journal of Orthopaedic Research, Biomaterials and Journal of Oral and Maxillofacial Surgery, totalizing 9 articles and open grey.

### 2.5. Data Collection Process

This research was carried out by methods used by previously calibrated researchers. The selection of articles and data collection was performed by a calibrated reviewers (T.J.L.-N.and L.P.F.). All titles and abstracts evaluated as eligible were separated and analyzed thoroughly to assess the titles and abstracts found, to obtain a concordance thesis value for the articles selected in both databases, and to reduce the possibility of bias in selecting articles. A meeting was required to reach consensus, and discrepancies were discussed and resolved by the third reviewer (J.F.S.Jr.).

### 2.6. Items to Be Extracted

The extracted data from each study were analyzed in an orderly manner, and the needed information was obtained in a standardized fashion. The following data were collected from the articles: authors, type of study, number of patients, age, sex, operated region of the body, type of screw, number of screws, surgery time, follow up, radiologic measures, functional recovery, laboratory values (mertal ion release), and complications. All data were collected by one reviewer (T.J.L.-N.) was then verified by another reviewer (L.P.F.). The data collection was entered in Excel spreadsheet (Excel, Microsoft, Washington, DC, USA).

### 2.7. Assessment of Study Quality and Risk of Bias

The ROBINS-I scale was applied for Non-Randomized studies of the effects of interventions [39]. This scale was developed by members of the Cochrane Bias Methods Group and the Cochrane Non-Randomised Studies Methods Group. For RCTs studies, the risk of bias in randomized trials was applied [35,40,41]. The online Robvis website (https://mcguinlu.shinyapps.io/robvis/) (accessed: 29 June 2020), was used to prepare responses for the seven areas presented in ROBINS-I and for the 5 domains presented on the ROB scale [40].

### 2.8. Types of Outcomes

#### 2.8.1. Primary Outcome

Evaluation of the clinical complications and failure rates of absorbable metal (Mg) plates/screws compared to Ti plates/screws used for bone surgeries

#### 2.8.2. Secondary Outcome

Analyses of radiologic measures, functional recovery, and laboratory results of the metal ions released.

#### 2.8.3. Additional Analysis

Sensitivity tests for subgroup analysis were performed in order to avoid the potential for heterogeneity considering, for example, possible differences in the different bone regions rehabilitated [42,43].

### 2.9. Meta-Analysis

#### Summary Measures

Quantitative data were grouped for some variables: the number of complications in patients who received absorbable Mg screws compared to the control group (Ti screws or bone grafting), the prevalence of severe complications, and failure in the Mg screw group was also calculated. This grouped information was evaluated for the event rate considering 95% CI. The number of treated patients who received surgical treatment was considered for data analysis (dichotomous data), which was used as a risk ratio (RR) [44,45].

A *p* value < 0.05 was considered significance. For event rate analyzes, the total number of patients who received absorbable Mg screws, and the total number of complications and failures were considered. The contribution weight of each study was also assessed. The Comprehensive Meta-Analysis software (Software version 3.0—Biostat, Englewood, NJ, USA) was used to construct the Forest plot [46].

## 3. Results

### 3.1. Qualitative Analysis

**Figure 1:**
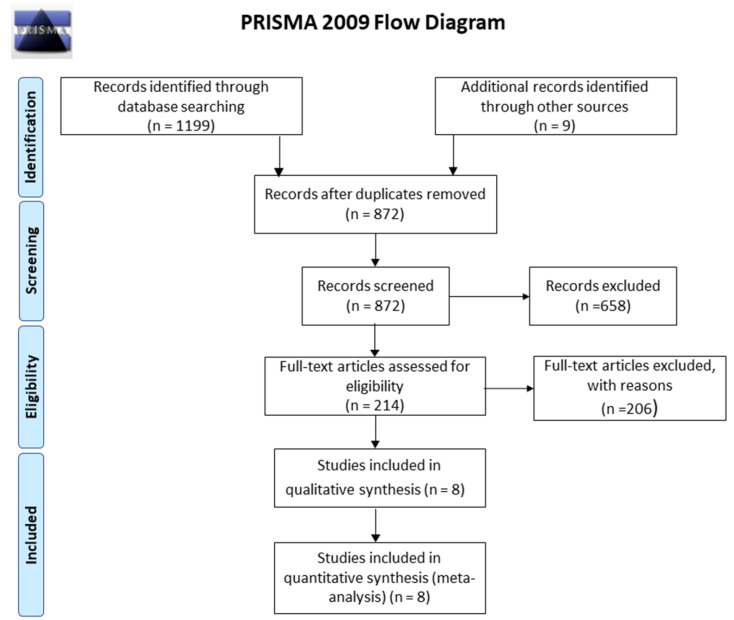
Flowchart of the studies selected for the systematic review.

In the initial search, 1199 articles were found according to the flowchart represented in Figure 1. After analysis of the inclusion criteria, eight articles were eligible, which 468 patients were included (control group or experimental group), with 230 screws of Mg. The age of patients included in all studies was 48.14 years [7,10,11,21,22,33,34,47]. The main results are summarized in Table 1.

### 3.2. Experimental Design

From eight studies selected, three were RCT studies [10,11,33], one were retrospective studies [34], two were case-control studies [11,22], and lastly, two were prospective case series [21,47]. All of these studies were published between 2013–2020. These studies are unclear about the specific location of the surgeries, but the report stated that surgeons were well-trained in their respective fields (Table 1).

### 3.3. Patient Selection

The studies analyzed reported various inclusion criteria for patient selection. Inclusion criteria were: age between 18 and 79 years, no medical contraindications, and surgical procedures that require fixation using screws [10,22]. The exclusion criteria included: patients with neurological diseases, surgeries in the same body region operated previously, allergies against the materials used for testing components of the screws [33,34] (Table 1).

### 3.4. Operated Region

Of the eight studies selected, six studies were related to the hallux region [7,10,21,22,33,34], one related to the treatment of necrosis of the femoral head [11] and last one related to mandibular condyle fracture [47]. All studies used screws for fixation. For each region operated, the most appropriate surgical procedure had a follow-up to evaluate the success of the treatment (Table 1).

### 3.5. Type of Screws

Five studies compared Mg and Ti screws related to the treatment of hallux fracture [7,10,22,33,34], one study used Mg screw compared to a control group with no graft fixation [11], and two studies had no control or comparison group [21,47] (Table 1).

### 3.6. Surgery Time

Only three studies determined the surgical time 40 min ± 9.1 (Mg) vs. 34 min ± 3.3 (Ti) [33]; 60.6 min (Mg) vs. 55.6 min (Ti) [34] and 35 min (Mg) vs. 34 min (Ti) [22]. The surgical times were closed for both groups (Table 1).

### 3.7. Radiologic Measures

All studies did some method of radiologic evaluation, and only one study described significant changes between the Mg and Ti group. In the Mg group, the authors classified 60% of the radiographs as satisfactory and 40% with some alteration, but these alterations were not specified. They only described areas of radiolucency, lytic areas, signs of plate or screw loosening, and bone resorption areas of demineralization, but no patients demonstrated any painful symptoms [34].

In the other studies, the radiographic evaluation showed no difference between the Mg and Ti group [7,10,22,33] (Table 2).

### 3.8. Follow-Up

The follow-up period ranged from 6 months to 36 months [10,33]. The mean postoperative follow-up was 12 months [7,11,21,22,34,47]. Despite the variation in follow-up time, the authors reported that it was enough to notice possible complications (Table 2).

### 3.9. Functional Recovery

All studies did some type of postoperative recovery assessment. Only one study reported positive results for Ti compared to the Mg group [7], whereas other studies showed similar results between the Mg group and the Ti group [10,22,33,34] (Table 2).

### 3.10. Laboratory Results

Only two studies reported Mg blood level assessment. Regarding the lab alterations, there was no difference between the groups [11,33] (Table 2).

### 3.11. Complications

The studies described the number of complications, one patient for Ti group with a screw head displayed, but the patient declined a re-operation surgery [34]. One patient in another study had a prominence of the screw, but no re-treatment was performed [22].

Regarding the evaluation of infections and postoperative healing, two studies reported patients with postoperative infection [7,34]. Klauser reported three patients had healing problems without signs of infection, two patients in the Mg group and one patient in the Ti group, and complications healed during the follow-up [34]. Thirteen patients had some type of infection of the surgical site, six from the Mg group and seven from the Ti group. The treatment for those infections ranged from the prescription of systemic antibiotic therapy to surgical treatment [7,22].

Regarding postoperative pain, one study [7] was very clear to discuss the pain evaluation between groups. In the Mg group, there were three cases of infection and one case of local pain. In the Ti group there were three cases of cellulitis; one case of regional pain; and one patient required implant removal. One study [5] reported that three patients for the Ti group had residual postoperative pain, while no patient in the Mg group reported any type of pain. In two other studies [11,47], no complications were related to the Mg group (Table 2).

## 4. Quantitative Analysis (Meta-Analysis)

### 4.1. Primary Outcomes

#### 4.1.1. Complications in the Absorbable Mg Screws vs. Control Group

**Figure 2:**
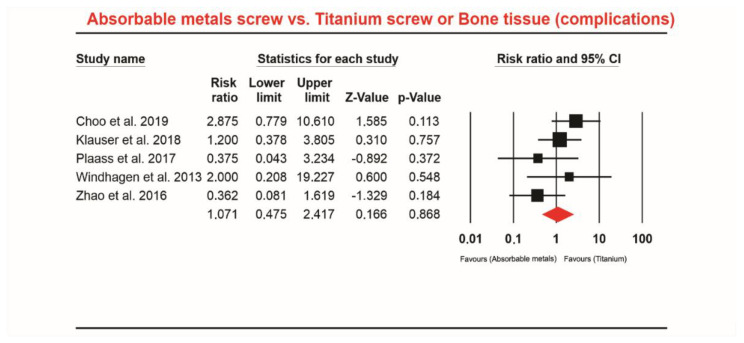
Forest plot for absorbable Mg-based screw vs. other materials.

Five studies [7,10,11,33,34] involving a total of 156 patients who received Mg screws identified 15 complications, and 213 patients received Ti screws or bone grafting, with 18 complications. The meta-analysis did not indicate a significant difference in this comparison (RR 1.071; 95% CI 0.475 to 2.417, *p* = 0.868, Figure 2). The heterogeneity was Q-value: 5.442, *p* = 0.245, I^2^ = 26.499.

#### 4.1.2. Complications in the Absorbable Mg Screw vs. Ti Screw Group and Hallux Valgus Deformity Surgery

**Figure 3:**
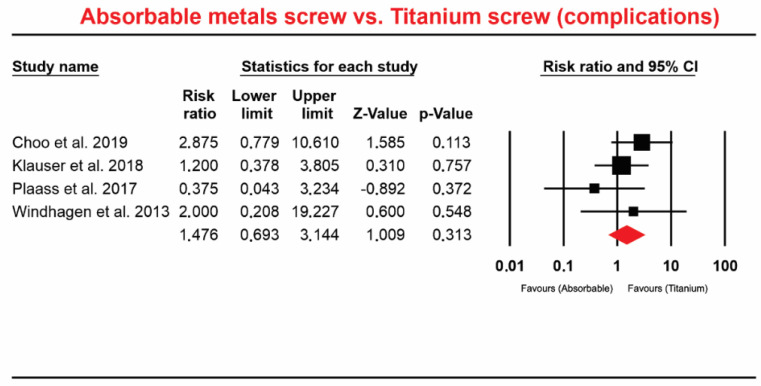
Forest plot for absorbable Mg-based screw vs. Ti screw (complication).

Four studies [7,10,33,34] involving a total of 145 patients who received absorbable Mg screws identified 13 complications and 188 patients received Ti screws in addition to being specifically for the region: Hallux valgus deformity, with 12 complications. The meta-analysis did not indicate a significant difference in this comparison (RR 1.476; 95% CI 0.693 to 3.144, *p* = 0.313, Figure 3). The heterogeneity was Q-value: 2.748, *p* = 0.432, I^2^ = 00.000.

#### 4.1.3. Failure in the Mg vs. Absorbable Screw Group Control (Ti Screw and Region: Hallux Valgus Deformity)

**Figure 4:**
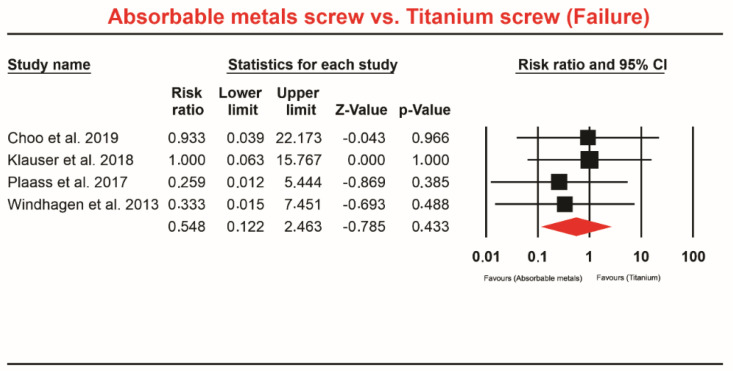
Forest plot for absorbable Mg-based screw vs. Ti screw (failure).

Four studies [7,10,33,34] involving a total of 145 patients who received absorbable Mg screws identified 1 failure and 188 patients received Ti screw or bone grafting, showing four failures. The meta-analysis did not indicate a significant difference in this comparison (RR 0.548; 95% CI 0.122 to 2.463, *p* = 0.433, Figure 4). The heterogeneity was de Q-value: 0.622, *p* = 0.891, I^2^ = 00.000.

#### 4.1.4. The Event Rate for Complications in Absorbable Mg Screw-In Operated Patients

**Figure 5:**
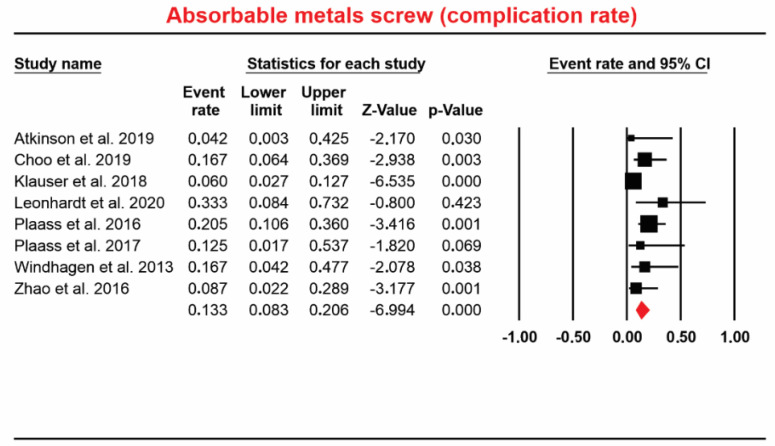
Forest plot for absorbable Mg-based screw (complication rate).

Eight studies [7,10,11,21,22,33,34,47] involving a total of 230 patients who received absorbable Mg screws identified 25 complications. Event rate data ranged from 8.3% to 20.6%. The overall pooled for event rate was 13.3% (random; 95% CI: 8.3% to 20.6%; Figure 5). Regions considered: Hallux valgus deformity, osteosynthesis of the mandibular condyle, osteonecrosis of the femoral head. The heterogeneity of the event rate for complications was considered to be *Q*-value: 9.448, *p* = 0.222, I^2^ = 25.907.

#### 4.1.5. Event Rate for Absorbable Mg Screw Failure in Operated Patients

**Figure 6:**
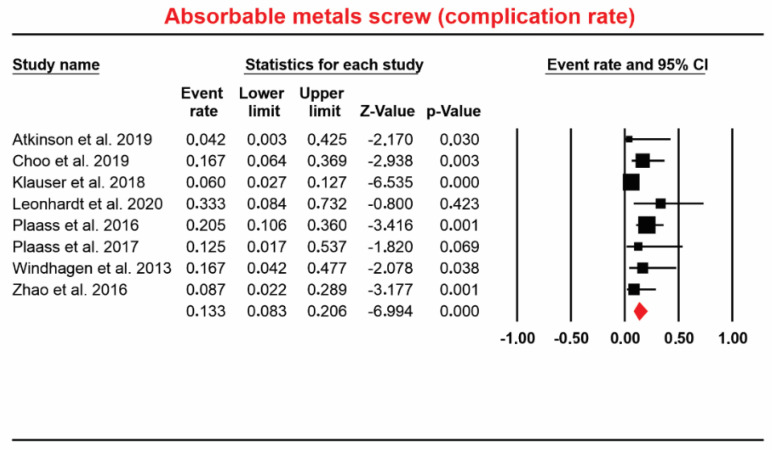
Forest plot for the absorbable Mg-based screw (failure rate).

Seven studies(7, 10, 21, 22, 33, 34, 47) involving a total of 207 patients who received absorbable Mg screws identified 3 failures. Event rate data ranged from 1.5% to 7.7%. The overall pooled for event rate was 3.4% (random; 95% CI: 1.5% to 7.7%; Figure 6). The heterogeneity of the event rate for complications was considered to be *Q*-value: 2.474, *p* = 0.871, I^2^ = 0.000.

### 4.2. Risk of Bias in the Studies

Heterogeneity was used using the Q method and the value of I^2^ was analyzed [45,48] heterogeneity above 75 (0–100) may reflect greater significance [45,49], we adopted analysis random for all meta-analyzes in order to reduce the potential for heterogeneity [50]. Particularities of the sample designs of each study were also evaluated and particularities of each forest plot were considered, considering, for example, specific analysis for hallux valgus deformity disregarding other regions, a control group containing only Ti screw was also considered, disregarding other materials.

### 4.3. Study Quality and Risk of Bias

#### Non-Randomized Studies

**Figure 7:**
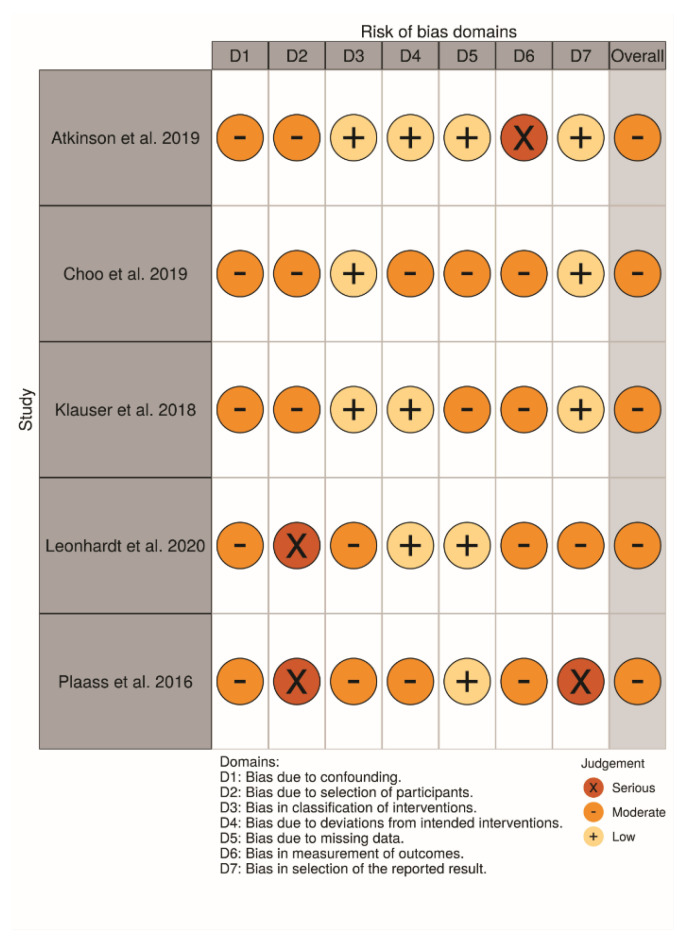
Risk of bias domains—ROBINS-I—Individual studies.

**Figure 8:**
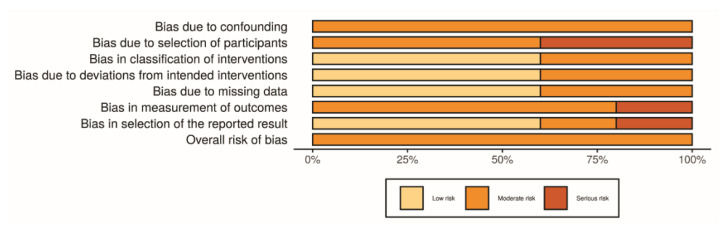
Risk of bias—Risk of bias domains—ROBINS-I—General information.

For non-randomized clinical studies [7,10,22,34,47], some studies either lacked the sample design, data were not proportional, or used retrospective data for test or control groups, which may all have influenced the outcomes. Limitations on the follow-up and lacking tomographic analysis for all the groups were also noted in some studies. Lastly, a lack of identification of the size of the screws in all studies, and only one study showed a sample size calculation. Figure 7 and Figure 8 show the main data on the risk of bias scale.

### 4.4. Randomized Studies

**Figure 9:**
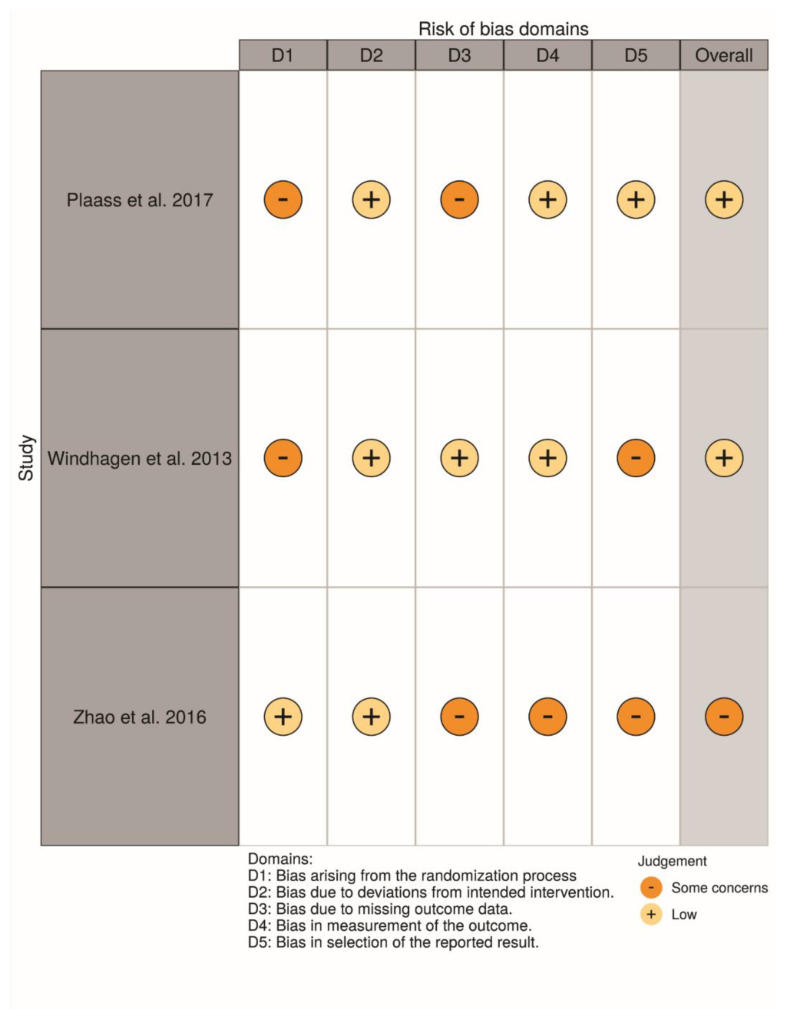
Risk of bias domains—ROB—Individual studies.

**Figure 10:**
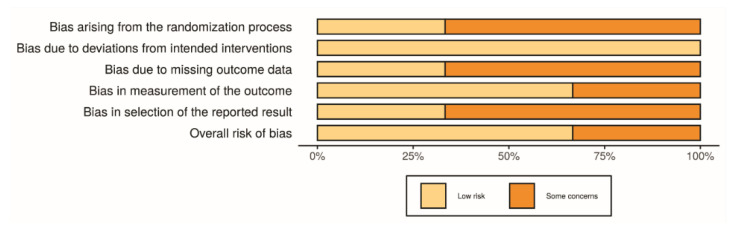
Risk of bias domains—ROB—General studies.

For randomized clinical studies [10,11,33], there was a limitation in the randomization methods. There was a lack of organization of the failures differently from the complications on each step of the evaluation. A short period of follow-up, or lack of information about systemic disorders or etiological factors also were noted. The main results for the evaluated domains are shown in Figure 9 and Figure 10, related to individual and general valuation, respectively.

Other methodologies information was also assessed, and it was noticed limitations related to data organization and identification of the sample size calculation (Table 3).

## 5. Discussion

This systematic review with a meta-analysis yielded that absorbable metals used for bone surgery, especially Mg alloys, were clinically useful and biologically acceptable compared to standard Ti implants. Of the 468 patients assessed in the studies selected, 230 Mg-alloys screws were used to stabilize a bone fracture [47], bone graft fixation [11], or correction of bone deformity [7,10,21,22,33,34]. Similar statistical data were noted for comparison between Ti and Mg screws (*p* = 0.433), as shown in Figure 1, showing acceptable biological responses for these applications.

Previous animal studies have found interesting results for absorbable metals in bone surgeries [34,51,52]. Most studies have suggested that Mg increases osteoblastic activity and has anti-inflammatory properties during the degradation process [26,53,54]. The vascular endothelial grown factor (VEGF), one of the most important factors to vascular proliferation, was significantly increased around Mg implants placed in bone marrow defects of rats [54]. The osteogenic and angiogenic properties with its degradation were achieved because of Mg ion and a co-enzyme of more than 200 enzymes from the organism which can be responsible to improve bone healing [55]. A few parts of Mg were distributed in the adjacent muscles, and other parts were metabolized by the kidney and liver without any postoperative blood chemistry alteration, as noticed by Windhagen et al., 2013 [33] and Choo et al., 2019 [7] (Table 2). The serum levels of calcium, Mg, and phosphorus were normal [11,26,33,56,57,58,59,60].

One of the concerns during Mg degradation is the hydrogen gas release. Some studies have speculated that the corrosion occurs with non-absorbable and absorbable metals. The particles around the bone and soft tissues could cause inflammation and bone resorption, including hypersensitivity reactions. However, none of the investigations found any evidence of allergenic effects in the presence of Mg-alloys [10,61,62]. One possible complication could be edema and emphysema during hydrogen gas release. Some cases indicated some radiographic signals of peri-implant gas radiolucencies. However, no clinical symptomatology existed, and the radiographic image change was solved at three months postoperatively [33,47,63,64].

In regards to the biomechanical properties, Mg screws have Young’ modulus very similar to bone tissue. However, over time, Mg could fatigue, especially when it requires a higher insertion torque. Recent studies have added other ions or metal particles, such as iron and zinc, to increase the mechanical resistance features [26]. Regardless, most of the clinical studies have found similar fatigue complications in the comparison between Mg and Ti materials for bone fixation [65,66]. One clinical strategy to decrease that complication is to use a countersink drill before screw insertion, since Mg screws are not self-drilling or self-tapping. Although this process may increase the time of surgery, the studies did not show a significant difference between Ti or Mg fixation, with only five-six minutes more time required for Mg surgeries [22,33,34] (Table 1).

The use of absorbable metals is very applicable in oral and maxillofacial surgery. For instance, in mandibular condyle fractures, when there is any instability of the temporomandibular joint complex during the postoperative period, an absorbable fixation system may avoid the development of some TMJ disorders or pathologies. Mg-MgHA/collagen-based scaffolds have been successfully used for sinus augmentation procedures. However, the result should be interpreted carefully as controls were not used for comparison [67]. The use of conventional Ti fixation in children is still controversial. Many studies suggested fixation removal after at least six months postoperatively, leading to more indirect costs related to the surgery and additional days for recovery of the patients. Therefore, an absorbable metal fixation becomes useful in children, with no necessity for a second surgery only to remove the fixation system. Although this study does not focus on an analysis of children, further research needs to be performed in the area [68,69,70,71,72].

In the orthopedic field, no significant differences were found between Ti and Mg screws used to bone fixation in all studies that assessed postoperative clinical parameters such as pain, walking/standing, and social interaction through standard scales demonstrated. In regards to better postoperative function, both treatments were effective [22].

Another important finding in the studies is the use of absorbable polymers, especially from the PLLA and poly-glycolic acid (PGA). Although the studies did not demonstrate significant differences in postoperative complications, absorbable polymers are mechanically weaker, and their degradation process through hydrolysis resulted in acidic elements, which can increase infections and bone resorption osteoclasts activation. The findings indicated that the use of PLLA and PGA might not be applicable for clinical applications, and the search for other degradable materials is warranted [14,73,74,75].

Several limitations were noticed in this systematic review as described above in the results section. These are fundamental to achieve an acceptable level of evidence for a clinical problem. The first one is the number of well-designed clinical investigations, including lack of clear methods of randomization, equality in the size of the sample for each experimental group (absence of sample calculations), limited follow-up, and different periods of follow-up among studies, limited organization of the failure’s assessment. Future studies should be designed following those standard parameters, especially through RCTs, involving control groups within the same study, and finally increasing the level of evidence of the results. Numerous studies have investigated Mg screws for fracture fixation of the maxillofacial and orthopedic bone surgeries. Future studies evaluating Mg plates for bioactivity properties should be warranted.

## 6. Conclusions

The use of Mg-based implants as absorbable metals for osteosynthesis show feasible applications in bone surgery procedures. There are no differences in a comparison between Mg-implants and conventional implants (Ti) with regards to biocompatibility or complication rates. Therefore, Mg-based implants should be considered for clinical applications in oral and orthopedic reconstructive surgery.

## Figures and Tables

**Table 1:** Quantitative data from selected studies.

Article	Type of Study	Number of Patients	Age (Mean in Years)	Sex (M or F)	Operated Region	Type of Screw	Number of Screws	Surgery Time (Min)	Follow Up (Months)
Windhagen et al.	Randomized Clinical Trial	26 (13 Mg; 13 Ti)	57.2 ± 7.2 Mg; 49.9 ± 16.5 Ti	11 m. 2 f. (Mg); 13 m. 0 f. (Ti)	Hallux	Mg; Ti	26 (13 (Mg); 13 (Ti))	40 ± 9.1 (Mg); 34 ± 3.3 (Ti)	6
Klauser	Retrospective	200 (100 Mg; 100 Ti)	52.34 (Mg); 50.87 (Ti)	NR	Hallux	Mg; Ti	200 (100 (Mg); 100 (Ti))	60.6 (Mg); 55.6 (Ti)	12.2 Mg; 11.7 Ti
Atkinson et al.	Case control study	36 (11 Mg; 25 Ti)	38 (Mg); 41 (Ti)	2 M., 9 F. (Mg); 2 M., 23 F. (Ti)	Hallux	Mg; Ti	36 (11 (Mg); 25 (Ti))	35 (Mg); 34 (Ti)	19 (12–30)
Choo et al.	Case control study	93 (24 Mg; 69 Ti)	54.5 ± 12 (Mg-Ti)	M.:1, F.:23 (Mg)	Hallux	Mg; Ti	93 (24 (Mg); 69 (Ti))	NR	12
Plaass et al.	Prospective case series	45 (Mg)	45.5 ± 10.6: 19.6–68.2	45 (2 m., 43 fe.)	Hallux	Mg	45	NR	12
Plaass et al.	Randomized Clinical Trial	14 (8 Mg; 6 Ti)	56 ± 8.9 (Mg); 52 ± 9.0 (Ti)	14 f. (Mg-Ti)	Hallux	Mg; Ti	14 (8 (Mg); 6 (Ti))	NR	36
Zhao et al.	Randomized Clinical Trial	48 (23 Mg; 25 C.)	30 ± 7 (Mg); 33 ± 8 (C.)	9 f./14 m. (Mg); 10 f./15 m. (c.)	femoral head	Mg	23 Mg	NR	12
Leonhardt et al.	Prospective case series	6 (Mg)	43.2: 30–66	4 m.; 2 f.	Mandibular condyle	Mg	6 Mg	NR	12

C: Control; Mg: Mg; Ti: Ti; M: Male; F:Female; Min: Minute.

**Table 2:** Qualitative data from the included studies.

Article	Radiologic Measures	Functional Recovery	Laboratory	Complications
Windhagen et al.	Correct placement of the implants and early signs of union and bone healing	all healed patients	No *	MgG(two patients had problems in healing) TiG (one patient had problems in healing; one patient had exposure of screw head)
Klauser	TiG. (All postoperative radiographs were satisfied.); Mg group: 60% of the radiographs as satisfy. and 40% with some alteration	There was no difference between groups (Mg group 3% vs. Ti group 4%)	NR	MgG (one broken screw; three patients with superficial infection; two patients with deep infection) TiG (one patient had prominence of the screw; four patients with superficial infections; one patient deep infection)
Atkinson et al.	No radiographic changes.	Mg—Improvement in postoperative results	NR	MgG (There were no post-operative complications of intraoperative technical)
Choo et al.	No radiographic changes of the screws in any group	The Ti group shows better results compared to the Mg group	NR	MgG (three cases of infection; one case of local pain) TiG (three cases of cellulite; one case of regional pain; one patient had implant removed)
Plaass et al.	The x-rays showed a significant improvement of all; Radiographic signs of bony healing	Improvement in postoperative results was observed	NR	Five patients (early implant disintegration, dislocation, radiolucency’s, or pain); two patients of early disintegration; seven patients showed functional problems after surgery)
Plaass et al.	There was no difference between the study groups regarding fracture repair	No difference regarding the rehabilitation of patients	NR	MgG no complications TiG (two patients’ pain during running; three patients had residual pain)
Zhao et al.	The tom. shows an increase in bone density compared to the control group	Favorable results for the Mg group compared to the CG	No *	There were no complications associated with the Mg group
Leonhardt et al.	Adequate repair of fractures was observed at 6-months postoperative tomography	All patients had experienced excellent restoration of their occlusion, and no revisions were required.	NR	No postoperative complications were reported

* NO: no change in the level of Mg in the blood; CG: Control Group; TiG: Ti Group; MgG: Mg Group. NR: Not is reported.

**Table 3:** Additional data verified.

Studies	Randomization	Sample Size Calculation	Suggestions	Limitation
Atkinson et al., 2019	No	No	Learning curve and multicentric studies	Sample not standardized before experiment
Choo et al., 2019	No	Yes	Higher sample	Largest sample; different screw sizes
Klauser et al., 2018	No	No	Higher sample	Short follow-up
Leonhardt et al., 2020	No	No	Higher sample and control group	Sample and comparison group
Plaass et al., 2016	No	No	Higher sample and follow-up	Reduced sample, short follow-up, absent of a control group
Plaass et al., 2017	Yes, but there was no description of the technique.	No	Learning curve	Reduced sample, data making some analyzes impossible
Windhagen et al., 2013	Yes. There was no description of the technique, but there was extern monitoring.	No	NR	Short follow-up, and some considerations related to the assessment of the screws and radiological images
Zhao et al., 2016	Sim	No	More multicentric studies	Consider etiological and other systemic factors
